# Vaginal lacerations during laparoscopic hysterectomy for endometrial cancer and local recurrence risk

**DOI:** 10.1016/j.gore.2024.101433

**Published:** 2024-06-16

**Authors:** Olivia Nicolais, Mackenzie Cummings, Tommy R Buchanan, Lea Moukarzel, Nicholas Cardillo, Elizabeth Burton, Mitchell I. Edelson, Mark S. Shahin

**Affiliations:** a Department of Obstetrics and Gynecology, Jefferson Abington Hospital, 1200 Old York Road, Price 109, Abington, PA 19001, United States; b Hanjani Institute for Gynecologic Oncology, Asplundh Cancer Pavilion of Sidney Kimmel Cancer Center, Thomas Jefferson University, 3941 Commerce Avenue, Willow Grove, PA 19090, United States

**Keywords:** Vaginal Laceration, Endometrial Cancer, Local Recurrence

## Abstract

**Objective:**

Laparoscopic surgery (MIS) offers equivalent oncologic outcomes as compared to open surgery, while causing lesser morbidity and resulting in a faster recovery. Vaginal extraction of specimens may cause vaginal or perineal lacerations (VL). The objective of this retrospective study was to assess local recurrence rates compared between cases with vaginal laceration (VL) or without vaginal lacerations (NL).

**Methods:**

We identified patients with endometrial cancer who underwent MIS between 2014 and 2018. We assessed the rate of local recurrence between patients in VL and NL cohorts. The study included all histologic subtypes and stages while benign final pathology, synchronous primaries or cases that required laparotomy for extraction were excluded.

**Results:**

338 MIS cases were evaluable of which 40 cases had a vaginal laceration during specimen extraction. There was no significant difference in age, race, presence of LVSI, stage, grade, histology or use of vaginal brachytherapy between cohorts. Cases with vaginal lacerations were significantly associated with a higher median BMI and larger uterine size. The VL cohort was more likely to have received adjuvant treatment. In early stage disease, more cases had non-endometrioid histology in the VL group and had increased incidence of chemotherapy and radiation use as well. There were no cases of isolated vaginal recurrence (0/40) in the VL group as compared to an incidence of 2 % (7/298) in the NL group with a relative risk of 0.48 (CI: 0.03–8.36, p = 0.62). There were 4 cases of pelvic recurrence (4/40) in the VL group and 2 cases in the NL group (2/298) with a relative risk of 2.13 (CI: 0.46–9.89, p = 0.34).

**Conclusions:**

In endometrial cancer cases, we did not observe a significantly increased risk of vaginal or pelvic recurrence after a vaginal laceration at the time of specimen removal.

## Highlights

• There appears to be no increase in vaginal recurrence of endometrial cancer in patients who had a vaginal laceration at time of uterine removal during laparoscopic surgery.
• There may be an increase in pelvic recurrence in patient who have a vaginal laceration at time of uterine removal during laparoscopic surgery.
• Further studies are needed to assess if removal of specimen in a bag changes risk of recurrence of endometrial cancer.

## 1 Introduction

Endometrial cancer is the most common gynecologic cancer in the developed world (Ferlay et al., 2015). In 2024 in the United States, the American Cancer Society estimates about 67,880 new cases of uterine cancer (including uterine sarcoma which represents about 10 % of cases) will be diagnosed and about 13,250 women will die from cancers of the uterus (“Key Statistics for Endometrial Cancer.” American Cancer Society, www.cancer.org/cancer/types/endometrial-cancer/about/key-statistics.html#:∼:text=In%20the%20United%20States%2C%20cancer,the%20uterus%20will%20be%20diagnosed. Accessed 2 Mar., 2024). Over the past 10 years, incidence has continued to increase and it is one of the few cancers with increasing mortality (“Key Statistics for Endometrial Cancer.” American Cancer Society, www.cancer.org/cancer/types/endometrial-cancer/about/key-statistics.html#:∼:text=In%20the%20United%20States%2C%20cancer,the%20uterus%20will%20be%20diagnosed. Accessed 2 Mar., 2024). Surgery is the standard of care in the treatment of endometrial cancer and stage is determined surgically (“Annual clinical conference: Diagnosis and treatment strategies for gynecologic cancer.” JNCI: Journal of the National Cancer Institute, June, 1985).

As minimally invasive surgery (MIS) became an option for treatment of endometrial cancer, questions arose as to the safety and outcomes for patients when compared to open surgery (OPS). Laparoscopic surgery for endometrial cancer was shown to offer equivalent oncologic outcomes compared to open surgery with the added benefit of less morbidity and faster recovery (Dinoi, 2023, Janda et al., 2017). In the LACE trial, progression free survival was studied in stage 1 endometrial cancer cases and showed no significant difference between laparoscopic versus open surgery for both disease free survival and overall survival (Janda et al., 2017).

While there are many advantages to hysterectomy performed via MIS for uterine cancer, vaginal extraction of specimens may cause vaginal or perineal lacerations (VL) (Chikazawa, 2022). To date there has been one study investigating risk factors for vaginal laceration with hysterectomy performed via MIS and only 15 of the 135 patients had endometrial cancer or endometrial hyperplasia (Chikazawa, 2022). There is another study from Canada which looked at uterine size at time of removal and risk of pelvic or distant recurrence which found that patients with a larger uterus were more likely to have a pelvic recurrence. This study did note that about 9 % of the patients had a vaginal laceration, but this was not noted on univariate or multivariate analysis in that study to increase recurrence and was not the primary focus of the study (Feigenberg, 2021). To our knowledge, no studies to date specifically report the impact of VL at the time of specimen removal during MIS on local recurrence after surgical treatment of endometrial cancer. The objective of this study was to assess local endometrial cancer recurrence rates in the vagina and/or pelvis between cases with or without VL at time of specimen removal in MIS cases for endometrial cancer. We hypothesized that VL would increase risk of local recurrence of endometrial cancer.

## 2 Materials and methods

This retrospective study was determined to be exempt by the Institutional Review Board of Thomas Jefferson University in Philadelphia, Pennsylvania. The requirement of informed consent was waived by the institutional ethics body due to the retrospective nature of the study. We included all endometrial cancer cases of all grades, stages and histologies managed via MIS between 2014 and 2018. The exclusion criteria were benign disease on final pathology, synchronous primaries, and cases that required a mini laparotomy for extraction.

Patient and disease characteristics including age, body mass index (BMI), obstetric history, histology, grade, stage, lymphovascular space invasion (LVSI), weight of uterus in grams, use of specimen bag, presence of and repair of vaginal or perineal lacerations, adjuvant treatment and vaginal or pelvic five year recurrence post surgery were extracted via chart review. VL was defined as any laceration noted in the operative report, regardless of whether or not a repair was performed. Of note, uterine manipulators are used universally for all laparoscopic hysterectomy cases at our institution.

For statistical analysis, Graphpad (r) online was used. Age was reported as mean with standard deviation. Given large distributions in BMI and uterine size, these were reported as median with range. The independent two sample *t*-test was used to compare continuous variables and the chi squared test was used to compare categorical variables. Relative risk of vaginal or pelvic recurrence was calculated with a 95 % confidence interval. For statistical analysis, P < 0.05 was considered significant.

## 3 Results

**Fig. 1:**
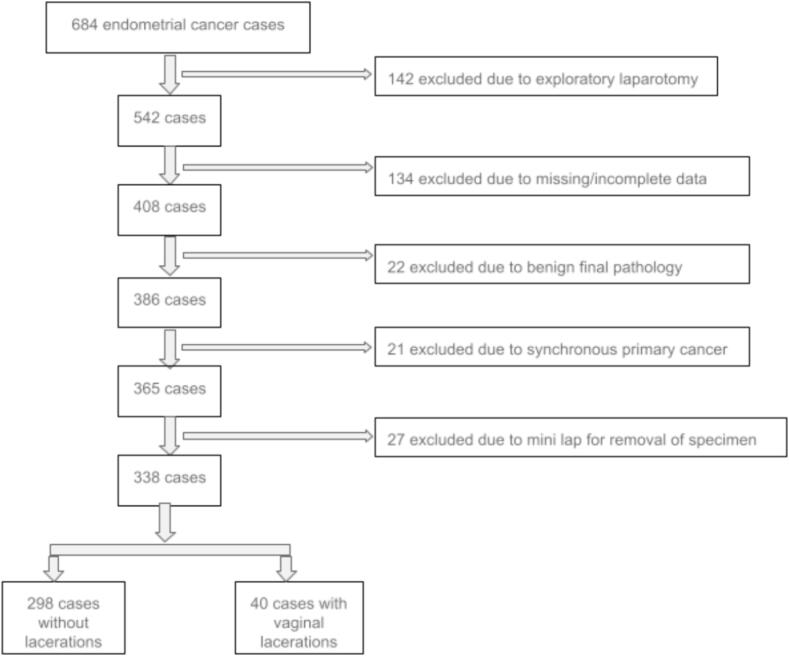

A total of 684 patients with endometrial cancer underwent surgery at Jefferson Abington Hospital between 2014 and 2018. Of these, 142 were excluded due to the case being completed via an exploratory laparotomy, 134 cases were excluded due to missing data or inadequate follow up documentation, 22 were excluded for benign findings on final pathology, 21 were excluded due to synchronous primaries and 27 cases were excluded due to need for a mini laparotomy for specimen removal leaving 338 cases to be included in the study. Of the 338 cases included, 298 cases were NL (88 %) and 40 cases were had a VL (12 %). **(**Fig. 1**)**. Baseline characteristics are reported in Table 1. There was no difference between groups (VL vs. NL) in age, race, presence of LVSI, grade, histology of endometrioid vs non endometrioid or use of vaginal brachytherapy (VBT). However, there was a trend towards more LVSI in the VL group (35.0 % vs 21.5 %, p = 0.08). The VL group had a statistically significantly larger BMI with a median of 33.6 (range 18.9–66.0) versus 32.3 (range 19.1–66.0) in the NL group (p = 0.03) although this difference is not likely clinically significant given the similar median values. Uterine size, as expected, was larger in the vaginal laceration group, median 150.5 g (range 49.0–462.0 g) versus 110 g (range 33.0–573.0 g) in the no laceration group (p= <0.01). The use of adjuvant external beam radiation therapy (EBRT) and chemotherapy were higher in the VL group (20 % versus 8.4 % for EBRT with p = 0.02 and 27.5 % versus 9.7 % for chemo with p= <0.01). In stage 1A disease alone, the number of cases receiving EBRT between groups was no longer significantly different (7 % vs. 2 %, p = 0.11). There was still more chemotherapy given in the stage 1A endometrial cancer VL group (15 % vs. 4 %, p = 0.04), likely due to a higher percentage of non-endometrioid histology in the VL group (27 % vs. 10 %, p = 0.007). Even with the higher use of EBRT and chemotherapy in the laceration group, the only cases of vaginal recurrence occurred in the NL group (7 cases of vaginal recurrence in the no laceration group and 0 cases of vaginal recurrence in the vaginal laceration group). In terms of pelvic recurrence there were 2 cases in the VL group (5 %) and 8 cases in the NL group (2 %). In regard to VBT, 10 patients in the VL group received adjuvant VBT (23 %) and 63 patients in the NL group received VBT (20 %). Higher pelvic recurrence rates were seen in patients treated with VBT in the VL group compared to the NL group (20 % vs. 5 %, p < 0.001) **(**Table 2**).** No patients that received VBT had a documented vaginal recurrence. There were 8 patients in the VL group treated with VBT who had no recurrence (80 %) and 61 patients in the no laceration group treated with VBT had no recurrence (97 %). The relative risk of vaginal recurrence in the VL group is 0.48 (CI 0.03–8.36, p = 0.62). The relative risk of pelvic recurrence in the VL group is 1.86 (CI 0.41–8.46,p = 0.42). In the cases with vaginal recurrence, 4 cases were stage 1A, 5 cases had endometrioid histology and 5 cases were grade 1 or 2. In cases that had a pelvic recurrence, 8 of the 10 cases were stage IA or 1B, 7 cases had endometrioid histology and 5 cases were grade 1 or 2 **(**Table 3**)**. Of the 338 patients, 262 (77 %) were type 1 (low grade endometrioid cancer). Although not statistically significant, the prevalence of type 1 patients who had a recurrence was 3 % versus 2 % (p = 0.09) in type 2 endometrial cancer (grade 3 endometrioid or non-endometrioid cancer).

**Table 1:** 

	No laceration group (n = 298)	Laceration group (n = 40)	P value
Age (mean)	64.7 (SD 10.7)	64.6 (SD 9.6)	0.99
Race	White 272 (91.3 %)	White 36 (90 %)	0.08
	Black 20 (6.7 %)	Black 3 (7.5 %)	
	Asian 6 (2 %)	Asian 1 (2.5 %)	
BMI (kg/m2) (median)	32.3 (range 19.1–66)	33.6 (range 18.9–66)	**0.03**
Uterine size (g) (median)	110 (range 33–573)	150.5 (range 49–462)	**<0.01**
#Vaginal deliveries	0 (85, 28.5 %)	0 (18, 45 %)	**<0.01**
	1 (58, 19.5 %)	1 (9, 22.5 %)	
	>/=2 (155, 52 %)	>/=2 (13, 32.5 %)	
LVSI	y (65, 21.8)	Y (14, 35 %)	0.06
Stage	1 (272, 91.3 %)	1 (32, 80 %)	0.06
	2 (6, 2 %)	2 (4, 10 %)	
	3 (20, 6.7 %)	3 (3, 7.5 %)	
	4 (0, 0 %)	4 (1, 2.5 %)	
Grade	1 (172, 57.8 %)	1 (20, 50 %)	0.25
	2 (70, 23.5 %)	2 (8, 20 %)	
	3 (56, 18.7 %)	12 (30 %)	
Histology	endometrioid (257, 86.2 %)	endometrioid (30, 75 %)	0.62
	Non endometrioid (41, 13.8 %)	non endometrioid (10, 25 %)	
Histology of stage 1A	endometrioid (204, 90 %)	endometrioid (19, 73 %)	0.007
	non endometrioid (21, 10 %)	non endometrioid (7, 27 %)	
Repaired with suture	0	34 (85 %)	n/a
VBT	63 (21.1 %)	10 (25 %)	0.55
EBRT	25 (8.4 %)	8 (20 %)	**0.02**
1A disease	5 (2 %)	2 (7 %)	0.11
Chemo (y/n)	29 (9.7 %)	11 (27.5 %)	**<0.01**
1A disease	10 (4 %)	4 (15 %)	**0.04**

**Table 2:** 

**VBT and Recurrence**		
	Laceration	No Laceration
Recurrence Vaginal	0 (0 %)	0 (0 %)
Recurrence Pelvic	2 (20 %)	3 (3 %)
No recurrence	8 (80 %)	61 (97 %)
Total patients treated with VBT	10 (23 %)	63 (20 %)

**Table 3:** 

**Vaginal recurrences (n = 7)**			
**Group**	**Stage**	**Grade**	**Histology**
NL	IB	3	serous
NL	IA	2	endometrial
NL	3A	2	endometrial
NL	IA	1	endometrial
NL	3C	2	endometrial
NL	IA	3	serous
NL	IA	2	endometrial

**Pelvic Recurrences (VL n = 2, NL n = 8)**			
**Group**	**Stage**	**Grade**	**Histology**
VL	IB	2	endometrioid
VL	IB	3	serous
NL	IA	3	endometrioid
NL	IIIB	3	clear cell
NL	IA	1	endometrioid
NL	IB	2	endometrioid
NL	IB	3	endometrioid
NL	IB	1	endometrioid
NL	IA	2	endometrioid
NL	IIIC	3	serous

In patients who had VL, four specimens were removed in a specimen bag (10 %) (uterine weight range 100 g −462 g) and there were no recurrences noted. No morcellation was performed in any case in which the specimen was removed in a specimen bag. Given relatively low use of a specimen bag and a low recurrence rate where a bag was used, no statistical analysis was completed for this group.

## 4 Discussion

### 4.1 Summary of main Results

While neither relative risk of vaginal or pelvic recurrence in either group is statistically significant, it is clinically of interest that there were no cases of vaginal recurrence in the VL group and the only cases of vaginal recurrence occurred in the NL group. However, in regard to pelvic recurrence, there were more cases in the VL group (4/40 cases) compared to the NL group (2/298 cases). One may hypothesize that due to expected increased manipulation to remove the uterus in the vaginal laceration group, perhaps there was seeding within the pelvis of disease through the cervix or Fallopian tubes. Given that there were increased rates of RT and chemo in the NL this could have affected our results given that patients who receive chemo and radiation likely had a higher baseline risk of recurrence which could explain the increased risk of vaginal recurrence.

Regarding removal of the uterine specimen in a bag, this study shows that this practice was not routinely done at our hospital between 2014 and 2018. Based on our observations, the practice of removing the uterus in a bag is done more frequently now than it was in the past, possibly due to ability to morcellate within the bag for easier removal or possibly based on extrapolation of increased recurrence rates in uterine cancer seen with power morcellation (Bojahr, 2015).

### 4.2 Results in the context of published Literature

There have been several studies assessing risk factors for recurrence of endometrial cancer. Recurrence is quite important in endometrial cancer because while overall prognosis in primary endometrial cancer is favorable, prognosis for recurrent cancer is poor (Huijgens and Mertens, 2013). A study by Huijgens et al in the Netherlands found that age, histological type and progesterone receptor positivity were independent risk factors for recurrence of endometrial cancer (Huijgens and Mertens, 2013). In our cohort, there was not a statistically significant difference in pelvic or vaginal recurrence between histological subtypes in those with and without lacerations (Table 4). This may be due to increased adjuvant treatment for patients who have grade 3 or non-endometrioid disease. We could not control for this association given both the retrospective nature of the study as well as NCCN recommendations for adjuvant therapy for those who have higher grade or non endometrioid histology. No patients treated with vaginal brachytherapy had a vaginal recurrence (Table 2).

**Table 4:** 

	No recurrence	Recurrence
Type 1 Endometrial Cancer	262 (78 %)	10 (3 %)
Type 2 endometrial Cancer	60 (17 %)	6 (2 %)

### 4.3 Strengths and Weaknesses

The strengths of this study include the large volume of endometrial cancer surgical cases as well as 5 year follow up at a single institution. Another strength is that all data including pathology and operative reports was reviewed by a single author throughout the course of the study to ensure uniformity in data collection. Limitations of the study include the low absolute number of cases with VL and small numbers of either vaginal or pelvic recurrence in both groups. Overall, survival and time to recurrence were not assessed in this study. Further multi institutional studies are needed in order to assess the risk of vaginal laceration on vaginal and/or pelvic recurrence.

### 4.4 Implications for practice and Future Research

Areas of future study include studies of specific sites of recurrence in open versus laparoscopic cases and to compare risk of vaginal site recurrence if a specimen is removed in a specimen bag or not. These findings have the potential to impact surgical planning, mode of specimen removal, change in surveillance or adjuvant treatment guidelines, and preoperative patient counseling. Future research should also include molecular classifications based on the ProMise study to see if this also has an effect of local recurrence risk. Future studies would also benefit from a multivariate analysis to better control for issues with laceration, histology, stage, and adjuvant treatment given.

## 5 Conclusion

In endometrial cancer cases, we did not observe a significant increased risk of vaginal or pelvic recurrence after a vaginal laceration at the time of specimen removal. This could be attributed to the higher rate of non-endometrioid histology in early stage disease, which resulted in higher rates of adjuvant chemotherapy or radiation treatment in the vaginal laceration group.

Mitchell Edelson- spouse is employee of Pfizer

Mark Shahin- Speaker’s Bureau and consultant for GSK, AZ and Merck, Speaker’s Bureau for Eisai and Immunogen, Expert witness (defense) for Saxton & Stump

## 6 Informed consent Statement

Informed consent was waived due to the retrospective nature of the study by the Jefferson IRB.

## Author Contributions

Olivia Nicolais, MD: Resident physician who conducted the background research, completed the data collection, performed statistics, primary author for the manuscript

Mackenzie Cummings, MD: Resident physician who assisted with writing the manuscript

Tommy R Buchanan, MD: Attending physician who assisted with statistics

Lea Moukarzel: Attending physician who assisted with writing the manuscript

Nicholas Cardillo: Attending physician who assisted with writing the manuscript

Elizabeth Burton: Attending physician who assisted with writing the manuscript

Mitchell I. Edelson: Attending physician who assisted with writing the manuscript

Mark S. Shahin: Attending physician principal investigator, assisted with writing the manuscript

## CRediT authorship contribution statement

**Olivia Nicolais:** Writing – original draft, Validation, Methodology, Formal analysis, Data curation, Conceptualization. **Mackenzie Cummings:** Writing – review & editing, Conceptualization. **Tommy R Buchanan:** Writing – review & editing, Supervision, Data curation. **Lea Moukarzel:** Writing – review & editing. **Nicholas Cardillo:** Writing – review & editing. **Elizabeth Burton:** Writing – review & editing. **Mitchell I. Edelson:** Writing – review & editing. **Mark S. Shahin:** .

## Declaration of competing interest

The authors declare the following financial interests/personal relationships which may be considered as potential competing interests: [Mark S. Shahin, MD. Speaker’s Bureau/ConsultantGSK. Speaker’s Bureau/ConsultantAZ. Speaker’s Bureau/ConsultantMerck. Speaker’s Bureau Eisai. Speaker’s Bureau Immunogen. Expert Witness (defense) Saxton&Stump. Mark S. Shahin, MD 03/24/2024 (e-signed)].

## References

[b0005] “Annual clinical conference: Diagnosis and treatment strategies for gynecologic cancer.” *JNCI: Journal of the National Cancer Institute*, June 1985.

[b0010] “Key Statistics for Endometrial Cancer.” *American Cancer Society*, www.cancer.org/cancer/types/endometrial-cancer/about/key-statistics.html#:∼:text=In%20the%20United%20States%2C%20cancer,the%20uterus%20will%20be%20diagnosed. Accessed 2 Mar. 2024.

[b0015] Bojahr B. (2015). Malignancy rate of 10,731 uteri morcellated during laparoscopic supracervical hysterectomy (LASH). Arch Gynecol Obstet..

[b0020] Chikazawa K. (2022). Risk factors associated with perineal and vaginal lacerations and vaginal removal in total laparoscopic hysterectomy. Gynecol Minim Invasive Ther..

[b0025] Dinoi G. (2023). minimally invasive compared with open surgery in high-risk endometrial cancer. Obstet. Gynecol..

[b0030] Feigenberg T. (2021). Factors associated with an increased risk of recurrence in patients diagnosed with high-grade endometrial cancer undergoing minimally invasive surgery: a study of the society of gynecologic oncology of Canada (GOC) community of practice (CoP). Gyn Oncology..

[b0035] Ferlay J., Soerjomataram I., Dikshit R. (2015). Cancer incidence and mortality worldwide: sources, methods and major patterns in GLOBOCAN 2012. Int J Cancer..

[b0040] Huijgens A.N., Mertens H.J. (2013). Factors predicting recurrent endometrial cancer. Facts Views vis Obgyn..

[b0045] Janda M., Gebski V., Davies L.C. (2017). Effect of total laparoscopic hysterectomy vs total abdominal hysterectomy on disease-free survival among women with stage i endometrial cancer: A randomized clinical trial. JAMA.

